# High‐Strength, Antiswelling Directional Layered PVA/MXene Hydrogel for Wearable Devices and Underwater Sensing

**DOI:** 10.1002/advs.202405880

**Published:** 2024-08-20

**Authors:** Shipeng Zhang, Fengmei Guo, Xue Gao, Mengdan Yang, Xinguang Huang, Ding Zhang, Xinjian Li, Yingjiu Zhang, Yuanyuan Shang, Anyuan Cao

**Affiliations:** ^1^ School of Physics and Laboratory of Zhongyuan Light Zhengzhou University Zhengzhou 450052 China; ^2^ Luoyang Institute of Science and Technology School of Intelligent Manufacturing Luoyang 471023 China; ^3^ School of Materials Science and Engineering Peking University Beijing 100871 China

**Keywords:** antiswelling, directional structure, PVA/MXene hydrogel, salting‐out, wearable sensor

## Abstract

Hydrogel sensors are widely utilized in soft robotics and tissue engineering due to their excellent mechanical properties and biocompatibility. However, in high‐water environments, traditional hydrogels can experience significant swelling, leading to decreased mechanical and electrical performance, potentially losing shape, and sensing capabilities. This study addresses these challenges by leveraging the Hofmeister effect, coupled with directional freezing and salting‐out techniques, to develop a layered, high‐strength, tough, and antiswelling PVA/MXene hydrogel. In particular, the salting‐out process enhances the self‐entanglement of PVA, resulting in an S‐PM hydrogel with a tensile strength of up to 2.87 MPa. Furthermore, the S‐PM hydrogel retains its structure and strength after 7 d of swelling, with only a 6% change in resistance. Importantly, its sensing performance is improved postswelling, a capability rarely achievable in traditional hydrogels. Moreover, the S‐PM hydrogel demonstrates faster response times and more stable resistance change rates in underwater tests, making it crucial for long‐term continuous monitoring in challenging aquatic environments, ensuring sustained operation and monitoring.

## Graphical abstract

Inspired by the Hofmeister effect, PM (PVA/MXene) hydrogel undergo salting‐out to achieve S‐PM hydrogel with excellent mechanical properties and crosslinking ability. Additionally, directional freezing introduces a layered structure, endowing the hydrogel with excellent tear resistance. The hydrogel exhibits a mere 6% change in resistivity after swelling and demonstrated superior sensing performance underwater.

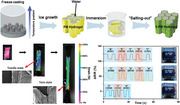

## 1 Introduction

The human body contains numerous load‐bearing soft tissues, including tendons, muscles, cartilage, and ligaments, all of which exhibited excellent mechanical properties.^[^ ^1^ ^]^ For instance, tendons, serving as connective tissues between muscles and bones, demonstrated remarkable anisotropy and high tensile strength, enabling them to undergo low plastic deformation during human movement.^[^ ^2^ ^]^ Moreover, their outstanding fatigue resistance rendered them indispensable load‐bearing components within biological systems. In comparison, synthetic hydrogels, due to their lower polymer chain density and heterogeneous polymer network, exhibited disordered porous structures and were prone to fragility and tearing during usage, thus impeding their applications in high‐strength and tough environments.^[^ ^3^, ^4^, ^5^, ^6^ ^]^ To address this issue, researchers have explored various methods to improve the energy dissipation mechanisms within hydrogels by establishing specific internal structures.^[^ ^7^, ^8^, ^9^, ^10^ ^]^ Common approaches included electrostatic interactions,^[^ ^11^, ^12^, ^13^ ^]^ hydrogen bonding,^[^ ^14^, ^15^, ^16^ ^]^ structural domain design,^[^ ^17^, ^18^ ^]^ and directional structure design.^[^ ^19^, ^20^ ^]^ For example, Zhu et al.^[^ ^21^ ^]^ utilized multinozzle 3D printing to achieve macroscopically tough hydrogel structures resembling titin domains, integrating these folded structural domains into a synthetic spider silk matrix, thus demonstrating significantly enhanced scalability and toughness. Liang et al.,^[^ ^22^ ^]^ on the other hand, combined bidirectional freezing and compression annealing to enhance the synergistic interactions between 2D layer‐structured hydrogels. This synergistic effect endowed the hydrogels with high ballistic energy absorption capacity, without compromising their high‐water content (85 wt%) and excellent flexibility, while also exhibiting extraordinary energy dissipation capabilities.

In addition to requiring an effective energy dissipation mechanism, high‐strength and tough hydrogels also need to possess good performance metrics in adverse environments. Particularly in underwater applications, hydrogels faced challenges due to their inherent 3D hydrophilic polymer network. In environments with high humidity, hydrogels were prone to experiencing osmotic pressure differentials, leading to the ingress of large amounts of water into their 3D network and resulting in swelling. This alteration in the mechanical properties of hydrogels could cause damage to devices.^[^ ^23^, ^24^, ^25^, ^26^ ^]^ Moreover, in sensor applications involving hydrogel devices, it was inevitable to introduce different ions into the hydrogel to enhance its conductivity. However, during hydrogel swelling, ions within the hydrogel unavoidably diffused out of the hydrogel network, leading to a decrease in its conductivity and sensing capabilities.^[^ ^27^, ^28^, ^29^ ^]^ To address these challenges, researchers needed to develop hydrogels with improved resistance to swelling and ion diffusion while maintaining their desired properties. This required innovative strategies in material design and synthesis to enhance the stability and functionality of hydrogels in various environmental conditions.

Improving the anti‐swelling capability of hydrogels has become a key research focus. Currently, researchers have altered the antiswelling capability of hydrogels through methods such as copolymerization of hydrophobic and hydrophilic monomers,^[^ ^30^, ^31^, ^32^ ^]^ polymer high‐density crosslinking, and ion complexation.^[^ ^33^, ^34^, ^35^, ^36^ ^]^ Enhancing polymer crosslinking density can increase the hydrogel's antiswelling capability by utilizing the Hofmeister effect, which involves manipulating the salt environment surrounding the hydrogel. Inspired by the Hofmeister effect, You et al.^[^ ^37^ ^]^ discovered that biocompatible hydrogels crosslinked by divalent anions exhibited stronger mechanical properties and lower swelling rates. Hydrogels crosslinked by divalent HPO_4_^2−^ anions showed excellent mechanical properties and anti‐swelling capability (16.7%). Zhang et al.^[^ ^38^ ^]^ discovered that polyelectrolytes could significantly enhance the mechanical properties of hydrogels through the Hofmeister effect. Introducing sodium polyacrylate anions into polyvinyl alcohol (PVA) hydrogels induced the aggregation and crystallization of PVA, thereby improving the mechanical properties of the double‐network hydrogels. Compared to polyacrylic acid, the tensile strength, compressive strength, Young's modulus, toughness, and fracture energy all saw substantial improvements. Although significant progress has been made in anti‐swelling research, it remains crucial to develop hydrogels that exhibit antiswelling while possessing energy dissipation mechanisms.

This study addressed these challenges by developing a layered, high‐strength, tough, and antiswelling PVA/MXene hydrogel using the Hofmeister effect, combined with directional freezing and salting‐out techniques. The hydrogel was prepared by mixing PVA and MXene, then applying directional freezing to form the layered PM hydrogel. The oxygen‐containing functional groups on the MXene surface not only formed hydrogen bonds with PVA but also, due to MXene's 2D structure, promoted the formation of an oriented network within the hydrogel, thereby enhancing its conductivity. Subsequently, the salting‐out process was employed to enhance the self‐entanglement of PVA, resulting in the S‐PM hydrogel with a tensile strength of up to 2.87 MPa. The unique layered structure and high strength of the S‐PM hydrogel allowed it to maintain excellent mechanical performance even when damaged, demonstrating significant potential in enhancing the mechanical stability of wearable devices. Additionally, the S‐PM hydrogel retained its structure and strength after swelling for 7 d, with only a 6% change in resistance. Importantly, its sensing performance improved after swelling, which was difficult to achieve with traditional hydrogels. These advancements were crucial for enabling long‐term continuous underwater monitoring, allowing operation and monitoring in challenging aquatic environments.

## 2 Results and Discussion

### 2.1 Design, Synthesis, and Structural Characterization of the S‐PM Hydrogels

**Figure 1:**
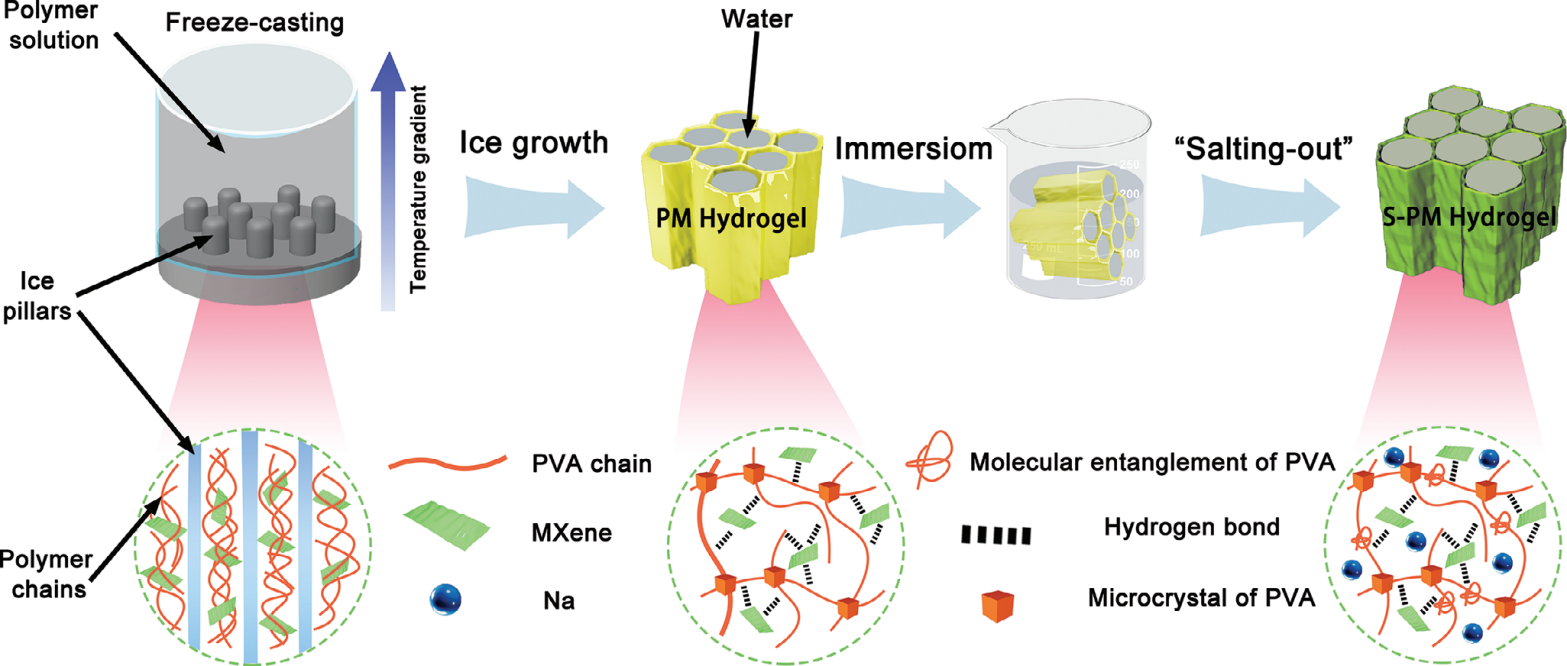
Illustrates the preparation of PM hydrogel and S‐PM hydrogel.

To obtain a conductive hydrogel with high mechanical strength and anti‐swelling properties, we synthesized a hydrogel, denoted as S‐PxMy, using a combination of directional freezing and salting out reactions. The preparation process of the hydrogel is illustrated in **Figure** **1**. S‐PxMy hydrogel possessed an oriented structure with dense layers, where x and y represent the volume ratio of PVA solution and MXene solution, respectively. The concentration of the PVA solution was 10 wt%, while the concentration of the MXene solution was 16 mg mL^−1^. Specifically, PVA was chosen as the primary matrix due to its tunable crystalline structure and biocompatibility.^[^ ^39^ ^]^ MXene, known for its excellent dispersibility, conductivity, and abundance of oxygen‐containing functional groups, was selected as the nanofiller to enhance the conductivity and mechanical properties of the hydrogel.^[^ ^40^ ^]^ The first step in preparing the S‐PM hydrogel involved directional freezing of the precursor solution containing PVA and MXene. This was achieved by placing the precursor solution on a copper block immersed in liquid nitrogen to control the direction of water crystallization. This step resulted in the formation of loosely cross‐linked PM hydrogel. During this stage, crystalline domains between PVA chains and hydrogen bonds between PVA and MXene were established, but significant entanglement between polymers was not observed, leading to an unstable cross‐linked network structure. Subsequently, the prepared PM hydrogel was immersed in a 1 m solution of dihydrate trisodium citrate. During this process, water gradually evaporated, reducing the distance between polymer layers and further increasing the degree of cross‐linking. This approach allowed us to create a hydrogel with enhanced mechanical properties, conductivity, and resistance to swelling, providing a promising platform for various applications.

### 2.2 Characterization Analysis of PM Hydrogel

**Figure 2:**
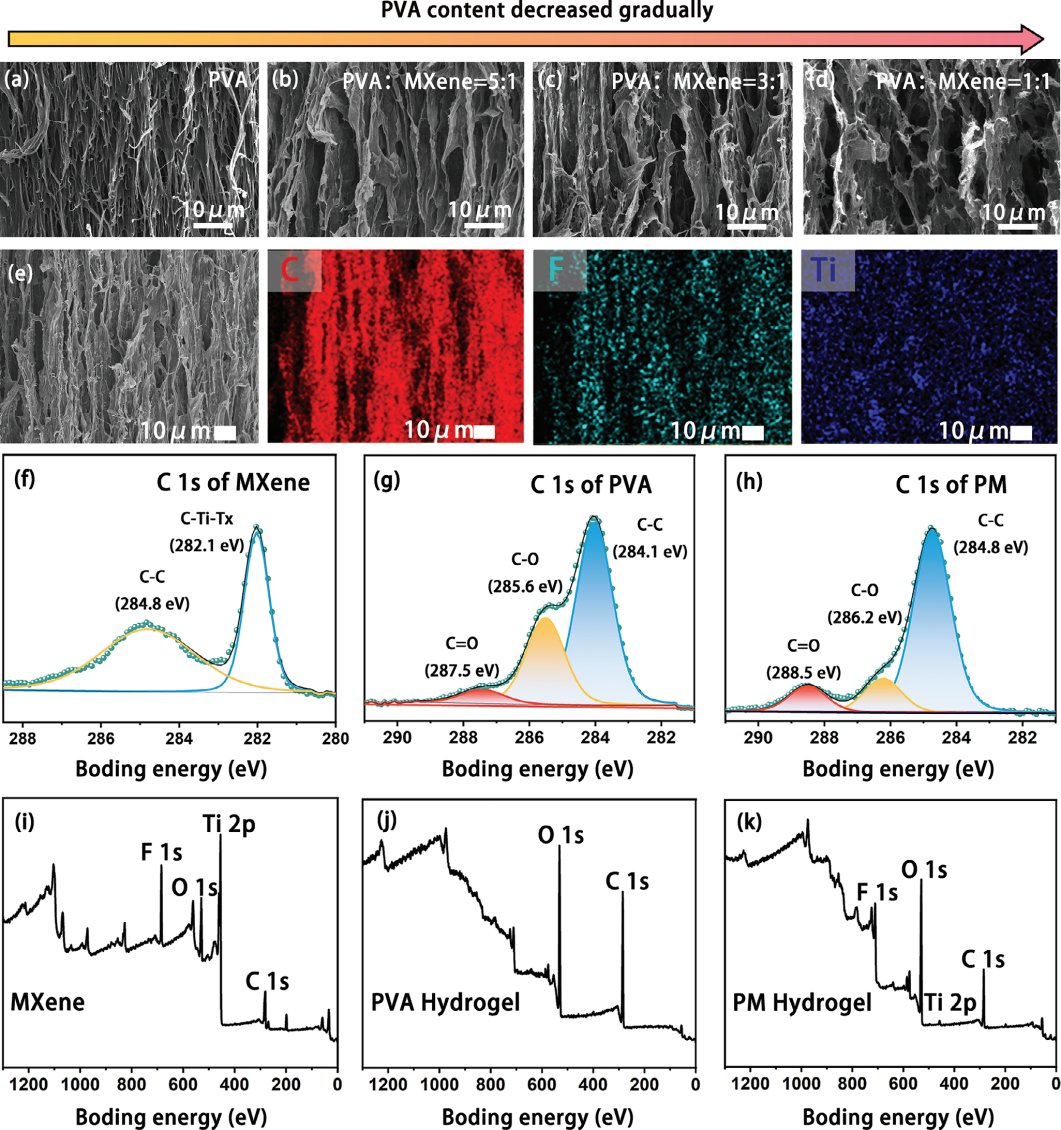
Structural analysis and characterization of PM hydrogel. a–d) SEM image of PVA:MXene = 1:0 hydrogel, PVA:MXene = 5:1 hydrogel, PVA:MXene = 3:1 hydrogel, PVA:MXene = 1:1 hydrogel. e) Elemental spectrum of PVA:MXene = 3:1 hydrogel. f) C 1s XPS spectrum of MXene. g) C 1s XPS spectrum of PVA hydrogel. h) C 1s XPS spectrum of PM hydrogel. i) Full spectrum of MXene. j) Full spectrum of PVA hydrogel. k) Full spectrum of PM hydrogel.

Scanning electron microscopy (SEM) images of samples with gradually decreasing PVA concentrations were presented in **Figure** **2**a–d. In Figure 2a, SEM image of the PVA‐only sample revealed a lamellar structure formed within the hydrogel, with small gaps between the layers, approximately 1–3 µm in size, controlled by temperature. SEM images depicted samples with PVA:MXene ratios of 5:1, 3:1, and 1:1, respectively (Figure 2b–d). As the PVA proportion decreased, the gaps between the layers increased. The interlayer spacing ranged from 2–6 µm at 5:1, expanded to 4–8 µm at 3:1, and reached nearly 10–12 µm at 1:1. This phenomenon arose from the introduction of MXene into the PVA solution, which reduced the entanglement between PVA chains, weakening their interactions. Subsequently, elemental distribution maps of the PM hydrogel were obtained via energy dispersive spectrometer (EDS) analysis (Figure 2e), revealing the lamellar structure distinctly, especially in the spectrum of carbon elements. The presence of MXene was further confirmed through testing the distribution of fluorine and titanium elements, demonstrating their uniform dispersion within the hydrogel, corroborating successful MXene incorporation.

In the X‐ray photoelectron spectroscopy (XPS) characterization of MXene, PVA, and PM hydrogels (Figure 2f–h), Figure 2f depicted the C 1s spectrum of MXene, with deconvolution peaks at 282.1 and 284.8 eV corresponding to C─Ti─Tx and C─C, respectively. In the full spectrum of MXene, the presence of Ti 2p and F 1s peaks confirmed the existence of functional groups such as ─F, ─OH, and ─O on the surface terminations of MXenes. These terminations were crucial for the material properties, such as hydrophilicity, conductivity, and interactions with other substances.^[^ ^41^ ^]^ Figure 2g presented the C 1s spectrum of PVA, with peaks at 284.1, 285.6, and 287.5 eV assigned to C─C, C─O, and C═O bonds, respectively. In the full spectrum of PVA, the C 1s peak primarily originated from the carbon─carbon and carbon─oxygen bonds in the main chain of the polymer. In the molecular structure of PVA, both the CH₂ and CH(OH) groups exhibited characteristic peaks within this range.^[^ ^42^, ^43^ ^]^ Figure 2h showed the C 1s spectrum of PM hydrogel, with an enhanced C═O bond at 288.5 eV and a decreased C─O bond intensity at 286.2 eV, suggesting the interaction between PVA and MXene. This provided evidence for the successful incorporation of MXene nanosheets into the hydrogel.^[^ ^44^ ^]^ Additionally, the presence of Ti 2p orbitals in the PM hydrogel spectrum confirmed the existence of MXene within the hydrogel. Further Fourier‐transform infrared spectroscopy (FTIR) analysis (Figure S2, Supporting Information) revealed that the ─OH and ─NH stretching vibration peaks of PVA corresponded to the 3500 to 3200 cm^−1^ range, and the main chain's C─H stretching vibration peak was at 2933 cm^−1^. The enhanced C─H bond in the PM hydrogel indicated that the incorporation of MXene increased hydrogen bonding. Overall, these findings highlighted the successful introduction and uniform dispersion of MXene in the PM hydrogel, along with the observed structural modifications.

### 2.3 Mechanical Properties of S‐PM Hydrogel

**Figure 3:**
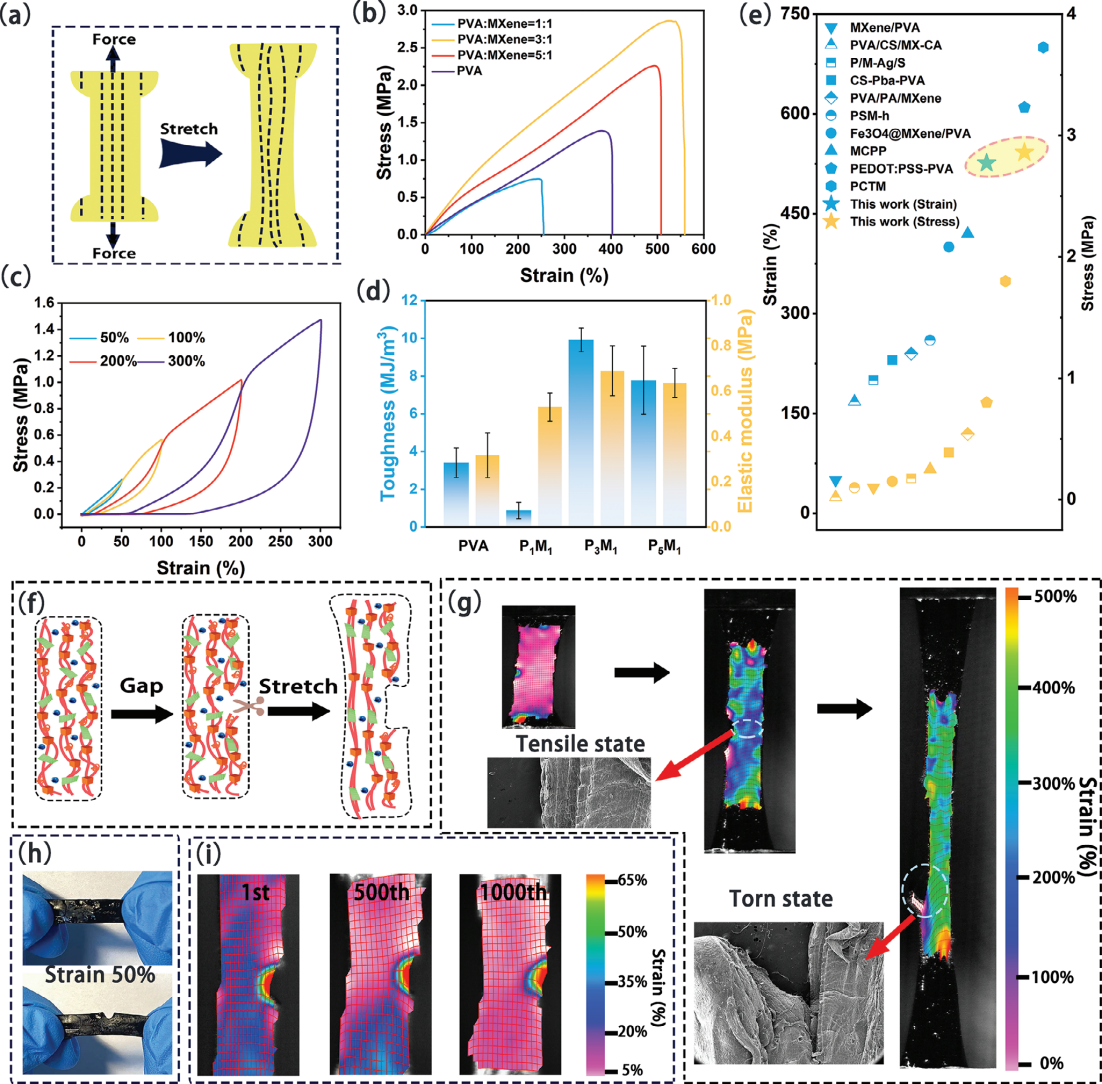
Mechanical performance testing of S‐PM hydrogel. a) Schematic diagram of tensile testing. b) Tensile stress of S‐PM hydrogel at different ratios. c) Tensile stress of S‐P_3_M_1_ hydrogel corresponding to different strains. d) Toughness and elastic modulus of S‐PM hydrogel at different ratios. e) Mechanical comparison of S‐PM hydrogel with other PVA and MXene composite hydrogels. f) Schematic diagram of tear resistance of S‐P_3_M_1_ hydrogel. g) DIC image showing tear resistance of S‐P_3_M_1_ hydrogel. h) Physical depiction of applied crack. i) Long‐term DIC image after crack application.

S‐PM hydrogel was subjected to stretching to compare the mechanical properties before and after salting out (**Figure** **3**a). Following salting out, the mechanical performance of the hydrogel significantly improved (Figure 3b), attributed to increased entanglement of PVA molecular chains and synergistic enhancement between MXene and metal ions. Particularly, the fracture strength of the S‐P_3_M_1_ hydrogel after salting‐out reached 2.87 MPa, with a maximum strain of 558%. The fracture strength was 168 times higher than before salting‐out. Prior to salting out, the maximum tensile stress and strain of P_3_M_1_ hydrogel were 17 kPa and 143%, respectively (Figure S3a, Supporting Information). The mechanical hysteresis curves at different strains illustrated that S‐P_3_M_1_ hydrogel followed the previous stress curve, experiencing substantial stress increases upon reaching the next strain point. This phenomenon may have stemmed from stress softening during strain, wherein the hydrogel initially stretched along the previous stress curve before significantly increasing stress at the next strain point (Figure 3c). Subsequent testing of the 100‐cycle tensile hysteresis curve at 100% strain (Figure S3b, Supporting Information) revealed stress reaching 0.64 MPa in the first cycle, decreasing to 0.52 MPa by the 10th cycle. The stress softening effect became more pronounced in extended cyclic testing, yet was notably mitigated in subsequent cycles due to the hydrogel's acclimatization to strain variations. Even after 100 cycles, the hydrogel maintained a stress of 0.50 MPa. Compression stress of S‐P_3_M_1_ hydrogel was measured, showing that at compression strains of 10%−50%, each compression stress curve followed the previous strain curve, indicating excellent elastic recovery capability within this range (Figure S3c, Supporting Information). Moreover, to verify the stability of elastic recovery, a long‐cycle compression test was conducted at a 30% compression strain. Throughout the 100 cycle compression loop, the hydrogel maintained robust elastic recovery (Figure S3d, Supporting Information).

Due to the salting out effect, S‐PM hydrogel significantly enhanced strength attributed to the formation of more self‐entanglement and hydrogen bonding within the hydrogel. Therefore, strength and elastic modulus of the hydrogel were analyzed through bar charts. As depicted in Figure 3d, the toughness of S‐P_3_M_1_ hydrogel reached 9.92 MJ m^−3^, while the elastic modulus reached 0.69 MPa. Subsequently, a comparison of strain and stress was conducted between S‐P_3_M_1_ hydrogel and other hydrogels reported in the literature (Table S1, Supporting Information). In the PVA and MXene hydrogel system, S‐PM hydrogel demonstrated outstanding tensile properties and exhibited high strength (Figure 3e).

S‐P_3_M_1_ exhibited high extensibility, strength, excellent toughness, and fatigue resistance, attributable to its multi‐level structure and toughening mechanisms. The toughening effect was primarily determined by the anisotropic arrangement of fiber bundles.^[^ ^45^ ^]^ During tensile testing, S‐P_3_M_1_ hydrogel displayed stepwise fracture, indicating its superior layered structure. As shown in Figure 3f, a shear notch was applied to the S‐P_3_M_1_ hydrogel to study crack propagation. In Figure 3g, the state of the sample at fracture was observed. It was found that the ordered alignment perpendicular to the crack path effectively hindered crack propagation. SEM and Digital Image Correlation (DIC) images revealed that under 50% tensile strain, the crack section of the S‐P_3_M_1_ hydrogel initially opened due to stress. However, as the strain increased, the stress at the crack section disappeared, gradually shifting to the non‐crack section, which then bore the pressure and eventually broke. At tensile strains above 400%, the strain in the non‐crack section increased while the strain in the crack section remained unchanged (Videos S1 and S2, Supporting Information). Additionally, shear‐tensile cyclic tests were conducted, and even after 1000 cycles of stretching to 50% strain, no crack propagation was observed in the S‐P_3_M_1_ hydrogel (Figure 3h,i). The outstanding mechanical performance of S‐P_3_M_1_ hydrogel also stemmed from its multiple reinforcement effects. Firstly, freeze casting concentrated amorphous PVA, forming stronger connections through hydrogen bonding. Subsequently, citrate substitution induced hydrophobic aggregation of PVA chains, forming more hydrogen bonds. Additionally, the binding of Na^+^ ions in sodium citrate solution to OH^−^ in PVA increased additional coordination bonds, with fracture energy even higher than hydrogen bonds. Hydrogen bonds and coordination bonds, as rigid, high‐functional cross‐linkers, also constituted more crystalline domains, significantly enhancing fracture energy, strengthening each layer, and endowing S‐P_3_M_1_ hydrogel with outstanding high strength and toughness.^[^ ^46^, ^47^ ^]^

### 2.4 Antiswelling Properties of S‐PM Hydrogel

**Figure 4:**
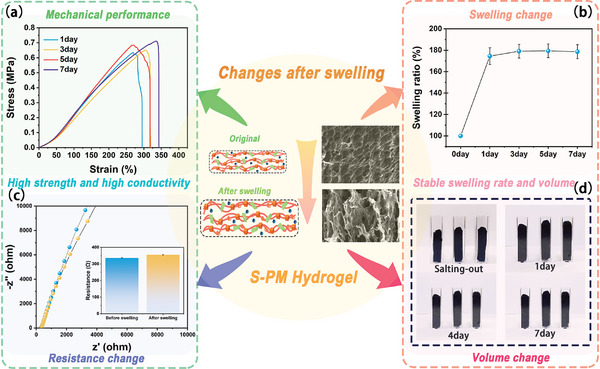
Swelling resistance performance testing of S‐PM Hydrogel. a) Mechanical changes after one week of swelling. b) Swelling ratio changes after one week of swelling. c) Changes in resistivity after one week of swelling. d) Changes in volume after one week of swelling.

S‐P_3_M_1_ hydrogel, owing to its unique fractal structure and high‐density crosslinking from salting out reactions, also demonstrated potential in resisting swelling. In **Figure** **4**a and S‐P_3_M_1_ hydrogel exhibited nearly identical mechanical properties after immersion in deionized water for 1, 3, 5, and 7 d, with mechanical strength remaining around 0.65 MPa. The decrease in mechanical strength compared to immediately after salting out was attributed to the disruption of the internal layered structure during the period from salting out to reaching water absorption equilibrium, as observed in the SEM images in Figure 4, where the layers became less dense and numerous pores appeared. However, after one day of water absorption, the damage ceased abruptly, and the mechanical strength stabilized and balanced. This stabilization was also evident in the swelling curve (Figure 4b), with the swelling ratio reaching 75% at equilibrium and remaining unchanged thereafter, with a swelling ratio of 78% on the seventh day. The stable swelling rate indicated that the anti‐swelling performance of the S‐P_3_M_1_ hydrogel was excellent, providing guidance for its long‐term underwater applications (Table S2, Supporting Information). It could also be seen from the swelling mass of the hydrogel that the mass did not change after the first day (Figure S4, Supporting Information).

Subsequently, the changes in conductivity (Figure 4c) and volume (Figure 4d) before and after swelling were tested. Using electrochemical impedance spectroscopy (EIS) impedance testing, the resistance of the S‐P_3_M_1_ hydrogel and its swollen state were measured, showing values of 333 and 353 Ω, respectively. The resistivity increased by only 6%, indicating that the hydrogel maintained good conductivity after swelling, which was highly beneficial for conducting sensing tests in water. The changes in volume were also clearly observable in physical photographs. Except for a noticeable volume change after one day of swelling, the hydrogel's size remained unchanged thereafter.

### 2.5 Electrical Properties of S‐PM Hydrogels before and after Swelling

**Figure 5:**
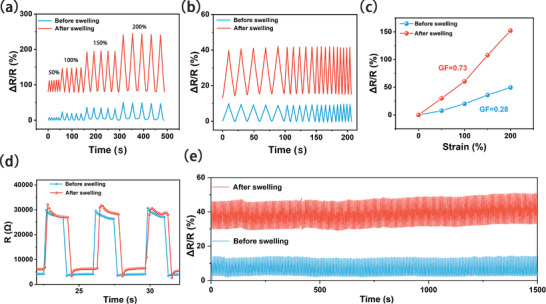
Electrical testing of S‐P_3_M_1_ hydrogel. a) Resistance change rate at different tensile strains before and after swelling. b) Resistance changes at different frequencies before and after swelling. c) GF of S‐P_3_M_1_ hydrogel before and after swelling. d) Break‐recovery response time of S‐P_3_M_1_ hydrogel before and after swelling. e) Electrical stability of S‐P_3_M_1_ hydrogel before and after swelling.

In addition to its outstanding mechanical properties, S‐P_3_M_1_ hydrogel also exhibited excellent conductivity, attributed to the introduction of MXene nanosheets during hydrogel synthesis, along with the incorporation of numerous free ions during the salting out process (Figure S5, Supporting Information). As shown in **Figure** **5**a, the S‐P_3_M_1_ hydrogel exhibited a stable resistance change rate across a strain range of 50% to 200% before and after swelling. The resistance change rate increased with larger strains. Figure 5b presented the frequency response of the S‐P_3_M_1_ hydrogel before and after swelling, showing stable resistance change rates at different frequencies. In Figure 5c, the gauge factor (GF) before and after swelling were statistically analyzed, revealing that the GF of the S‐P_3_M_1_ hydrogel improved after swelling from 0.28 to 0.73. This enhancement in sensing performance postswelling was relatively rare (Table S3, Supporting Information). This change was attributed to the hydrogel absorbing more water during the swelling process, which released some entanglements between the PVA chains. This led to an increased contact area between the MXene nanosheets in the hydrogel, forming more pores. The influx of water provided additional conductive pathways, whose combined effects further enhanced the sensitivity.

The electrical self‐healing properties of hydrogels played a crucial role in sensor applications, particularly in flexible electronic devices and biomedical sensors. With this in mind, we tested the electrical self‐healing performance of the S‐P_3_M_1_ hydrogel both before and after swelling. It was found that the S‐P_3_M_1_ hydrogel, after being disconnected and reconnected, took only 0.28 seconds to restore its conductivity. This excellent electrical self‐healing performance further underscored the potential of the S‐P_3_M_1_ hydrogel in sensing applications (Figure 5d). Additionally, in the final long‐term cycling test, the S‐P_3_M_1_ hydrogel demonstrated outstanding cyclic stability over 500 cycles both before and after swelling. Before swelling, the S‐P_3_M_1_ showed a resistance change rate of approximately 10% at 50% strain, while the swollen S‐P_3_M_1_ exhibited a resistance change rate of around 24% at 50% strain. Both maintained stable resistance change rates at the beginning and end of the cycles (Figure 5e). This also confirmed that the swollen S‐P_3_M_1_ hydrogel not only retained good cyclic stability but also did not lose its electrical self‐healing ability.

In Figure S6 (Supporting Information), the response and relaxation times of the S‐P_3_M_1_ hydrogel before and after swelling were compared. The unswollen S‐P_3_M_1_ hydrogel had response times of 700 ms in air and 600 ms in water, while the swollen hydrogel had response times of 500 and 300 ms, respectively. This demonstrated that the swollen hydrogel had superior response times and was more suitable for underwater sensing. Additionally, long‐cycle tests under 50% compression strain underwater were conducted on the hydrogel before and after swelling. The results showed that the swollen hydrogel had better stability underwater, attributed to the increased water content within the S‐P_3_M_1_ hydrogel after swelling, making it more compatible with underwater applications.

### 2.6 Sensing Properties of S‐PM Hydrogels in Air and Water

**Figure 6:**
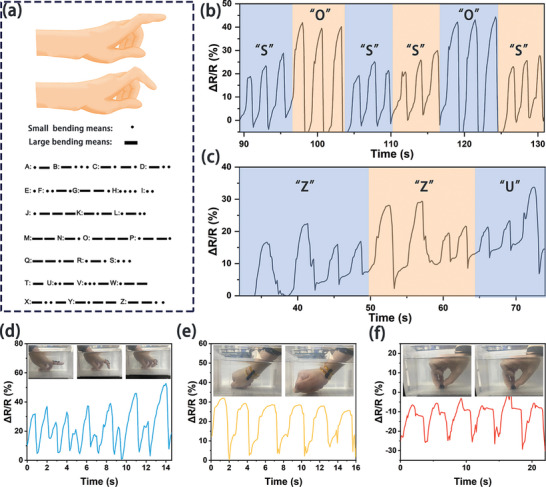
Sensing capability of S‐P_3_M_1_ hydrogel. a) Schematic diagram of Morse code. b) Recognition of SOS signal through Morse code. c) Recognition of ZZU signal through Morse code. d) Underwater detection of finger bending signal. e) Underwater collection of elbows bending signal. f) Underwater collection of pressing signals.

Due to the excellent mechanical and electrical properties of S‐P_3_M_1_ hydrogel, it showed promising application potential in integrated wearable sensors. As shown in **Figure** **6**a, the S‐P_3_M_1_ hydrogel accurately recognized Morse code. We defined different degrees of finger bending as dots and dashes and designed tests for the signals “SOS” (Figure 6b) and “ZZU” (Figure 6c). These tests showcased the hydrogel's ability to recognize different signals through repeated use, highlighting the superiority of the sensor (Video S4, Supporting Information).

Moreover, the S‐P_3_M_1_ hydrogel demonstrated good stability in water environments, making it suitable for sensing or monitoring tasks in such conditions, such as water quality monitoring and underwater communication. This made it ideal for applications requiring prolonged immersion or underwater operations. Based on these advantages, its underwater motion monitoring capability was tested. It was found that the S‐P_3_M_1_ hydrogel could monitor finger bending underwater (Figure 6d), reflecting the degree of bending by detecting changes in resistance. Additionally, tests involving wrist bending (Figure 6f) and pressing (Figure 6e) showed stable resistance change rates, indicating that the S‐P_3_M_1_ hydrogel possesses excellent and stable sensing performance.

### 2.7 Human–Machine Interaction Capability of S‐PM Hydrogel

**Figure 7:**
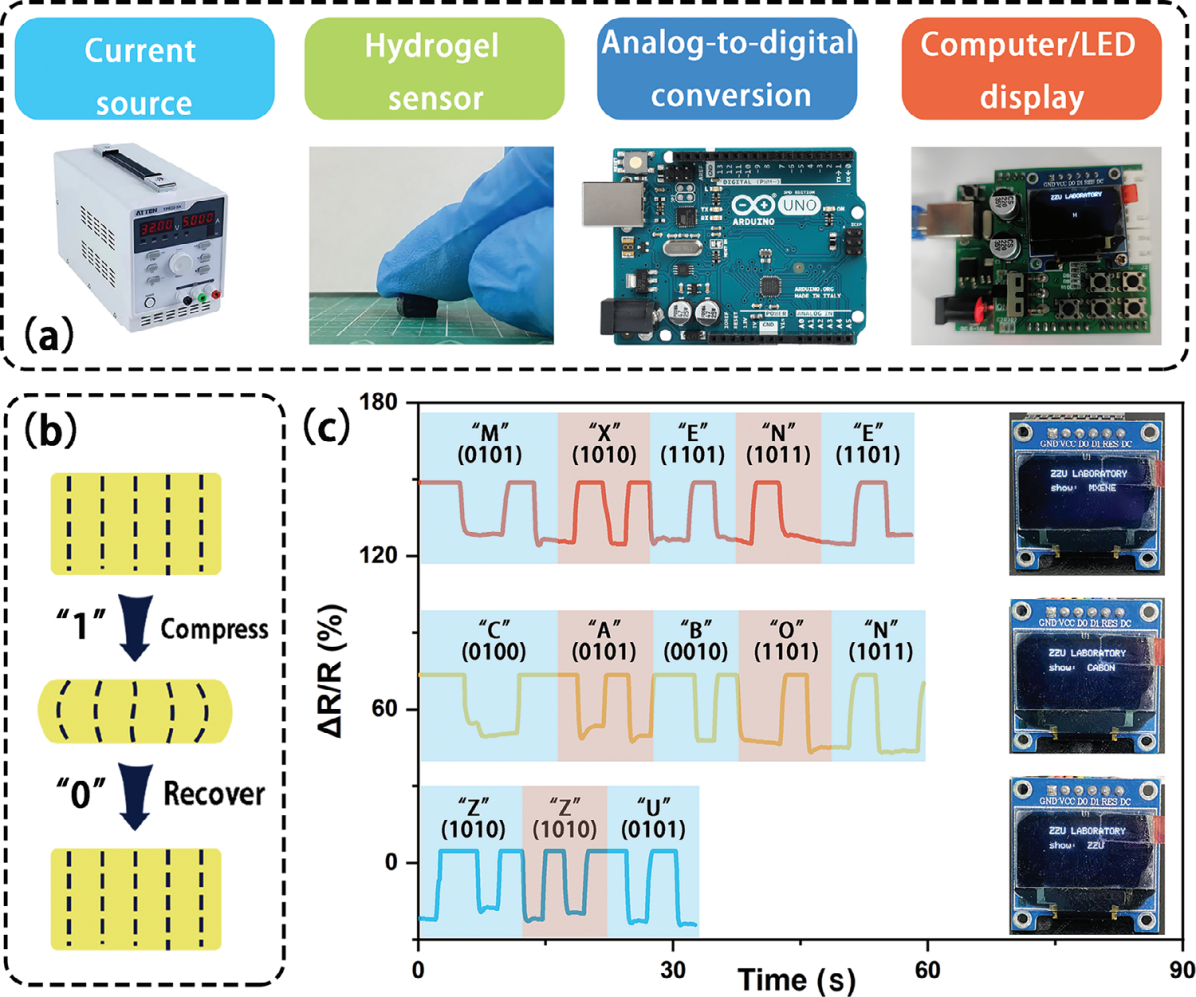
Human–machine interaction capability of S‐PM hydrogel. a) Human–machine interaction module and its physical diagram. b) Schematic diagram defining 0 and 1 codes. c) Letters required for LED display controlled by pressing.

Due to the outstanding mechanical and sensing performance of S‐P_3_M_1_ hydrogel, it also held promise for controlling screen displays, offering application potential in wearable human–machine interface electronic products. To achieve precise control over LED displays, a 16‐bit high‐precision analog‐to‐digital conversion chip was employed to convert the analog pressure signals into digital signals. This was accomplished through a computer interface module for transmission, ensuring high synchronization between the pressure signals and LED displays. The entire data flow process was illustrated in **Figure** **7**a, involving DC power supply, hydrogel sensor pressure, analog‐to‐digital conversion module, and LED display. In Figure 7b, differentiation between 0 and 1 codes was demonstrated, where pressure corresponds to 1 and recovery to 0.

This study controlled the supply current of the DC source to match different sensitivity materials and strain signals, while observing real‐time current changes for adjustments, ensuring the generation of relative electrical signals with each press. Real‐time linear mapping technology, combined with high‐precision module sampling and short‐distance transmission characteristics, achieved linear analog motion with close to zero latency, distinguishing it from existing control methods (Video S3, Supporting Information). Experimental results demonstrated that S‐P_3_M_1_ hydrogel could not only control the simple display of letters on LED screens but also dynamically change the codes to display different combinations of letters, such as ZZU, CARBON, and MXENE in real‐time (Figure 7c).

## 3 Conclusion

In summary, the S‐PM hydrogel, possessing high mechanical strength and excellent resistance to swelling, demonstrated remarkable performance in wearable sensor devices and underwater sensing. The hydrogel was prepared by directional freezing of a mixed solution of PVA and MXene in an environment with a temperature gradient, resulting in an anisotropic and layered hydrogel structure. The incorporation of MXene not only introduced oxygen‐containing functional groups on its surface and formed hydrogen bonds with PVA but also facilitated the formation of a directional network of 2D sheet‐like structures within the hydrogel, enhancing its structural strength and conductivity. Subsequently, further salting‐out of the hydrogel in a solution of trisodium citrate was performed to further enhance its mechanical strength. The resulting S‐PM hydrogel exhibited a tensile strength of 2.87 MPa without sacrificing its extensibility (fracture strain of 558%). The oriented arrangement of the hydrogel endows it with remarkable resistance to crack propagation, which greatly prolongs its working life in flexible wearable devices and underwater sensor devices, making it suitable for challenging operating environments. These findings provide insights for the exploration and development of high‐strength, high‐flexibility, and high‐conductivity hydrogels, thereby promoting the advancement and application of integrated wearable sensor technology.

## 4 Experimental Section

### Materials

Polyvinyl alcohol (PVA, grade 1799, with a degree of hydrolysis of 98% to 99%) was purchased from Shanghai Maclin Corporation, China. Disodium citrate dihydrate (C_6_H_5_Na_3_O_7_ 2H_2_O) was obtained from Tianjin Chemiou Chemical Reagent Co., Ltd., China. Concentrated hydrochloric acid (HCl, 9 m) was sourced from a Beijing Chemical Factory, China. Ti_3_AlC_2_ was procured from Jilin Yiyi Technology Co., Ltd., China. Lithium fluoride (LiF, 99.9%) was acquired from Adamas (Shanghai, China). All chemicals were used without further purification, and all experiments were conducted using deionized water.

### MXene Aqueous Solution

Initially, 2 g of Ti_3_AlC_2_ powder and 2 g of LiF were added to 40 mL of 9 m HCl solution. The mixture was stirred at 35 °C for 22–34 h. Subsequently, the resulting product was washed with deionized water until the pH of the solution reached 7. The supernatant obtained after washing was collected. Prior to utilization, the collected supernatant was subjected to filtration for concentration measurement. The stratification of MXene was observed by TEM characterization (Figure S1, Supporting Information).

### Preparation of PM and S‐PM Hydrogels

Initially, 5 g of PVA were dissolved in 45 mL of deionized water. Subsequently, 15 mL of PVA aqueous solution were mixed with 5 mL of MXene aqueous solution with a concentration of 16 mg mL^−1^. The two solutions were then stirred for 2 h. The resulting mixture was centrifuged to remove bubbles, and then transferred to a mold measuring 10 × 10 × 50 mm^3^. The mold was placed on a copper block immersed in liquid nitrogen, and subjected to three freeze‐thaw cycles to form PM hydrogel.

The prepared PM hydrogel was immersed in a 1 m solution of disodium citrate dihydrate for 12 h to form S‐PM hydrogel.

### Characterizations

The chemical structures of MXene and nanocomposite hydrogels were analyzed using FTIR (Bruker VERTEX 70 V) in the range of 400–4000 cm^−1^ and XPS (Thermo Fisher, Nexsa). The microstructures of MXene and composite hydrogels were characterized using SEM (Zeiss Auriga) and TEM (JEOL JEM‐2100).

### Mechanical Performance Testing

The mechanical properties of the hydrogel were evaluated using an electronic universal material testing machine (Instron 5943). Dumbbell‐shaped hydrogels (total length of 40 mm, gauge length of 20 mm, width of 8 mm, thickness of 2–3 mm) were tested at a stretching rate of 0.5 mm s^−1^. stress (*σ*, in kPa) and strain (*ε*, in %) were calculated using σ  =  *F*/*A*_0_ and ε  =(*L* − *L*_0_) *100%, where *F* is the load (N), *A*_0_ is the initial cross‐sectional area of the hydrogel (m^2^), and *L* and *L*_0_ are the lengths under load and initial lengths, respectively.

### Electrical Performance Testing

The hydrogel was cut into strips with a length of 20 mm, width of 7 mm, and thickness of 7 mm for EIS testing using an electrochemical workstation (CHI660E). The conductivity K (S m)^−1^ was calculated using the formula *K*  =  *L*/R*S, where *L* is the distance between adjacent electrodes (m), *R* is the resistance obtained from EIS measurements (Ω), and *S* is the cross‐sectional area (m^2^).

Using an electronic universal material testing machine and a digital source meter (Keithley 2400), the current variation of the conductive hydrogel with strain was recorded under a constant voltage of 1 V. The corresponding resistance was calculated based on Ohm's law. Then, the resistance change rate was determined using the formula Δ*R*/*R*  =  (*R* − *R*_0_)/*R*_0_*100%, where *R_0_* and *R* are the resistances with and without applied strain, respectively. The gauge factor (GF) was obtained using the formula GF  =  (Δ*R*/*R*)/ε, where *ε* represents strain, and GF is the strain sensitivity of the sensor.

### Swelling Resistance Testing

The swelling resistance of the hydrogel was quantitatively determined by recording the mass increase ratio of the hydrogel samples in deionized water at room temperature (24 °C). The initial mass of the hydrogel was measured, and it was then immersed in deionized water until it reached equilibrium, with no further change in mass observed. The mass increase ratio (*W*) was calculated using the formula *W*  =  *M*/*M*_0_, where *M* and *M_0_* represent the equilibrium mass of the hydrogel after immersion in deionized water and the initial mass, respectively.

### Informed Consent

All wearable sensing tests were done with my consent.

## Conflict of Interest

The authors declare no conflict of interest.

## Supporting information

Supporting Information

Supplemental Video 1

Supplemental Video 2

Supplemental Video 3

Supplemental Video 4

## Data Availability

The data that support the findings of this study are available from the corresponding author upon reasonable request.
