# Supplementary material for: High‐Strength, Antiswelling Directional Layered PVA/MXene Hydrogel for Wearable Devices and Underwater Sensing
Source: Adv Sci (Weinh). 2024 Aug 20;11(39):2405880. doi: 10.1002/advs.202405880 (PMC11496995; doi:10.1002/advs.202405880)
Supplement: Supplementary file 1 — Supporting Information [file ADVS-11-2405880-s001.pdf]

ADVANCED SCIENCE
Open Access

Supporting Information

for *Adv. Sci.*, DOI 10.1002/advs.202405880

High-Strength, Antiswelling Directional Layered PVA/MXene Hydrogel for Wearable Devices and Underwater Sensing

Shipeng Zhang, Fengmei Guo, Xue Gao, Mengdan Yang, Xinguang Huang, Ding Zhang, Xinjian Li, Yingjiu Zhang, Yuanyuan Shang and Anyuan Cao*

Supporting information:

High-strength, anti-swelling directional layered PVA/MXene hydrogel for wearable devices and underwater sensing

Shipeng Zhang[1], Fengmei Guo[1], Xue Gao[2], Mengdan Yang[1], Xinguang Huang[1], Ding Zhang[1], Xinjian Li[1], Yingjiu Zhang[1], Yuanyuan Shang[1]*, Anyuan Cao[3].

1 School of Physics and Laboratory of Zhongyuan Light, Zhengzhou University, Zhengzhou 450052, China

2 School of Materials Science and Engineering, Peking University, Beijing 100871, China

3 Luoyang Institute of Science and Technology, School of Intelligent Manufacturing, Luoyang 471023, China

Corresponding authors. E-mail address: yuanyuanshang@zzu.edu.cn (Y. Shang)

Total number of pages: 6

Total number of figures: 6

Total number of videos: 4

Total number of tables: 3

Table of Contents

Figure S1……………………………………………………………….……..……...S2
Figure S2……………………………………………………………..……......…S2
Figure S3……………………………………………………………..……......…S3
Figure S4…………………………………………………………….....……...S3
Figure S5…………………………………………………………….....……...S4
Figure S6…………………………………………………………….....……...S4
Video S1…………………………………………………………….............…S5
Video S2……………………………………………………………..……...…S5
Video S3…………………………………………………………………………S6
Video S4…………………………………………………………………………S6
Table S1…………………………………………………………………… ………S7
Table S2…………………………………………………………………… ………S7
Table S3…………………………………………………………………… ………S7

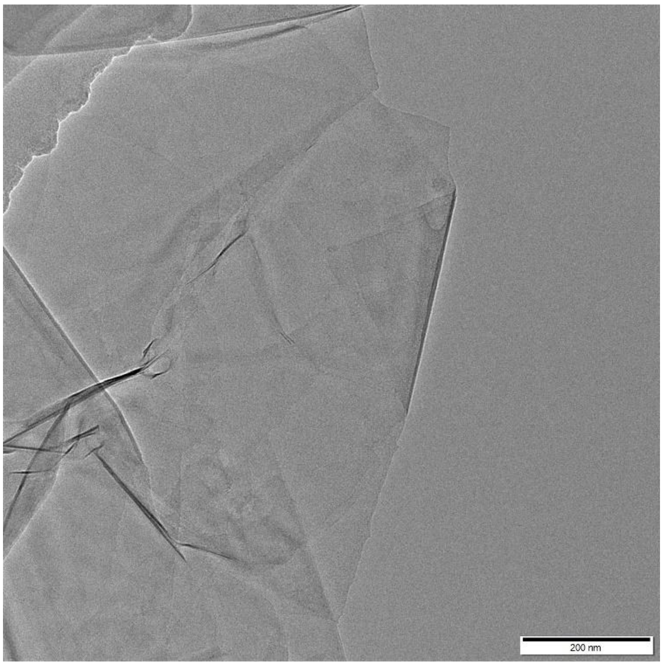

Figure S1 TEM image of MXene.

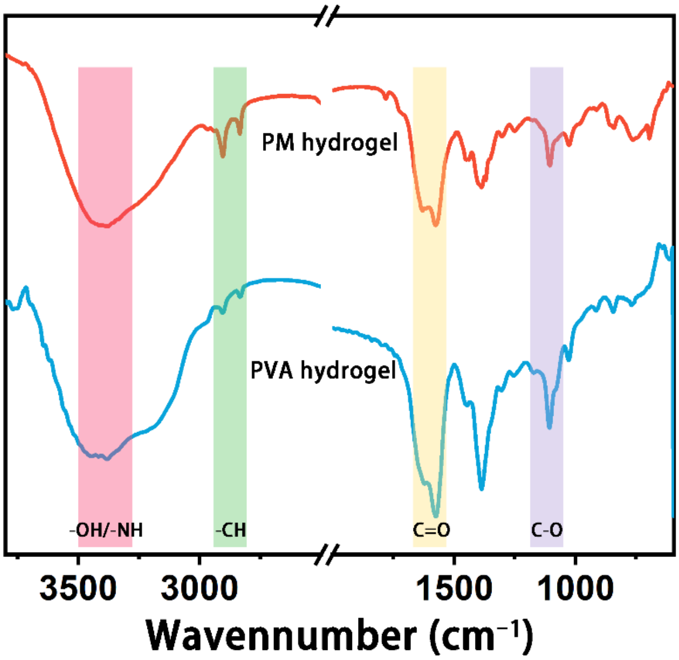

Figure S2 FTIR spectra of PVA and PM hydrogels.

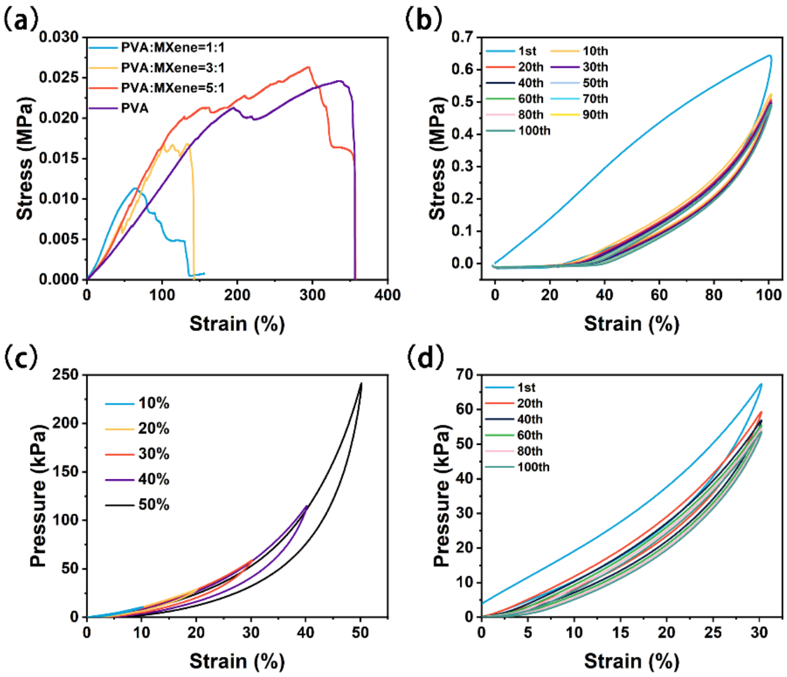

Figure S3 Mechanical property supplement. (a) Tensile stress-strain curve of PM hydrogel (b) Long cycle curve of 100% tensile strain of S-PM hydrogel (c) Compressive strain curves of S-PM hydrogel from 10% to 50% (d) Long cycle curve of 30% compressive strain of S-PM hydrogel.

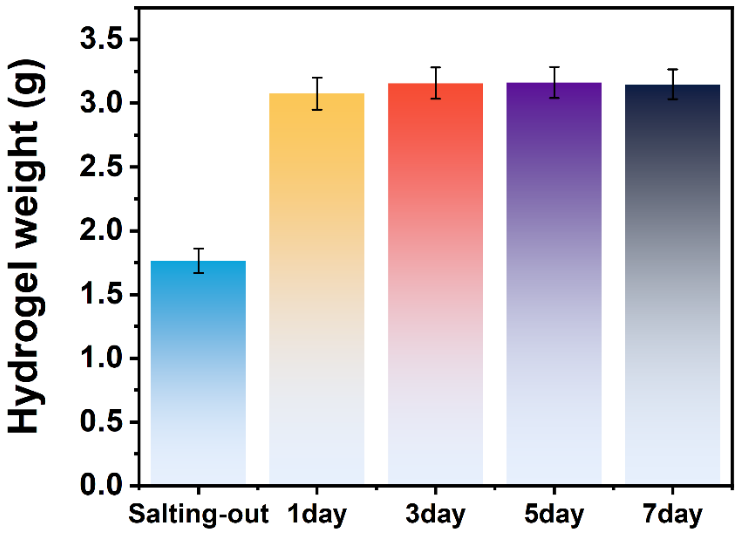

Figure S4 Mass change during swelling week.

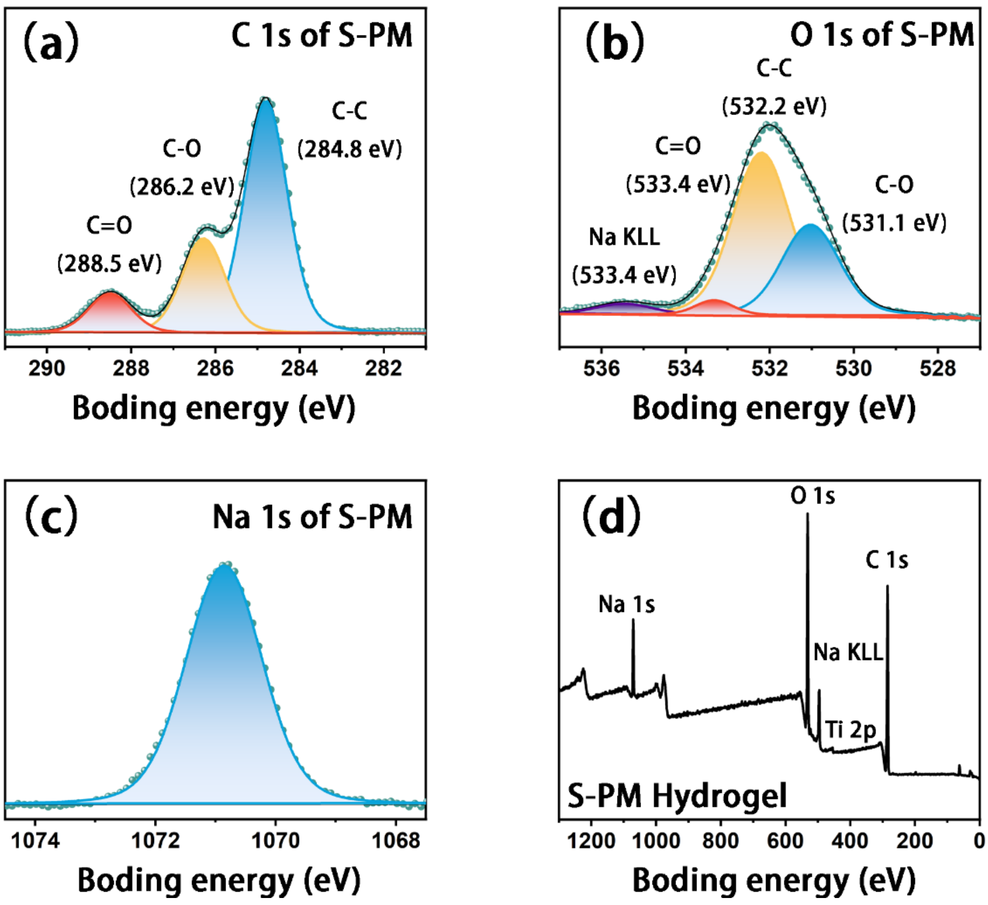

Figure S5. Characterization of PM hydrogel. (a) C 1s XPS spectrum of S-PM hydrogel. (b) O 1s XPS spectrum of S-PM hydrogel. (c) Na 1s XPS spectrum of S-PM hydrogel. (d) Full spectrum of S-PM hydrogel.

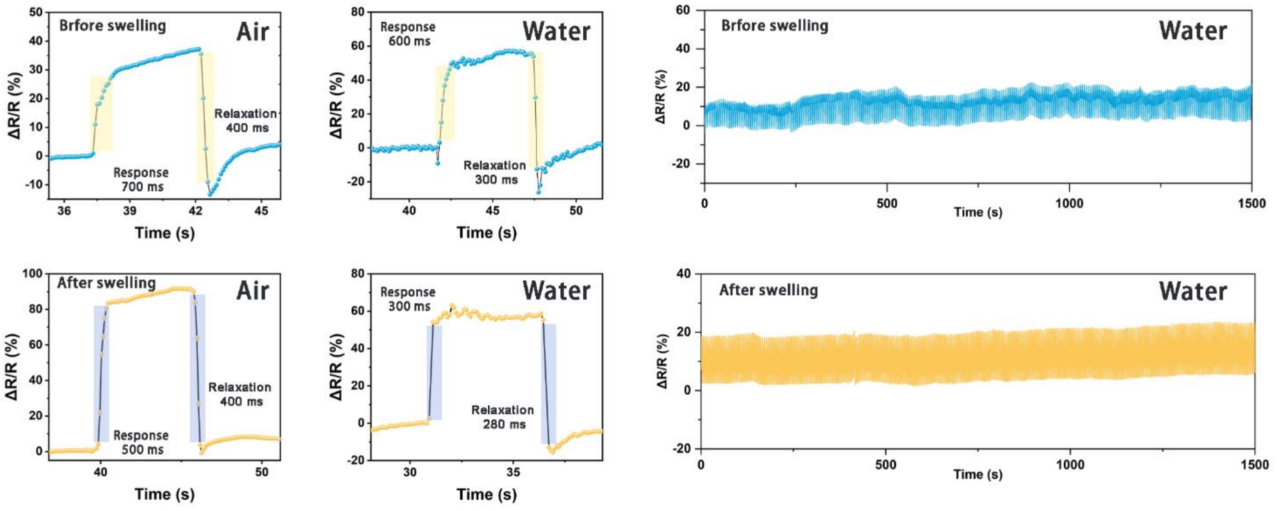

Figure S6. The response and relaxation times of the S-P_3M_1 hydrogel before and after swelling in air and underwater, as well as the long-cycle stability underwater before and after swelling.

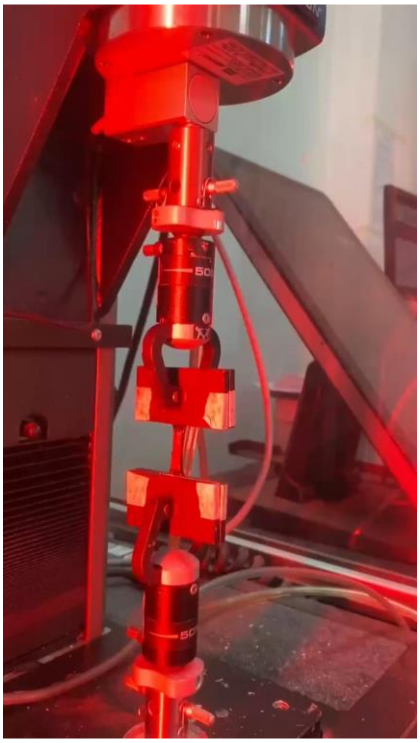

Video S1 S-P_3M_1hydrogel stretching video.

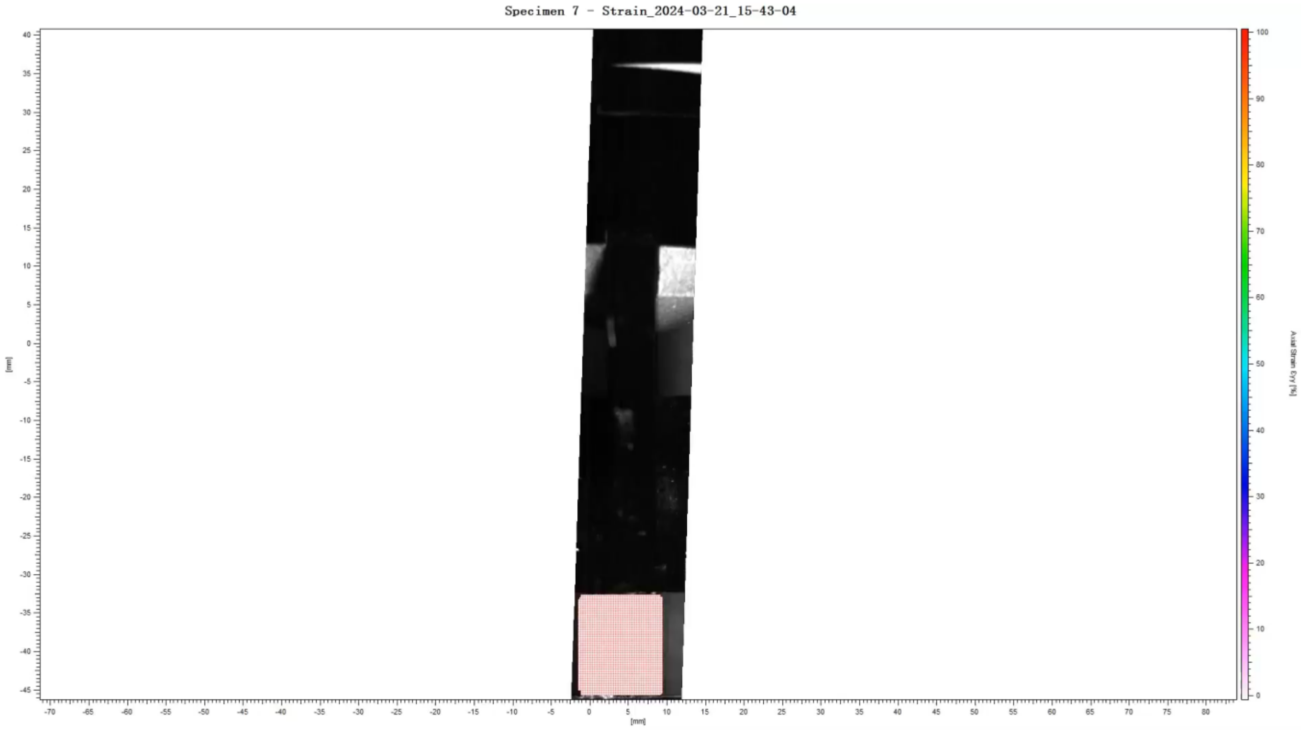

Video S2 S-P_3M_1hydrogel DIC test.

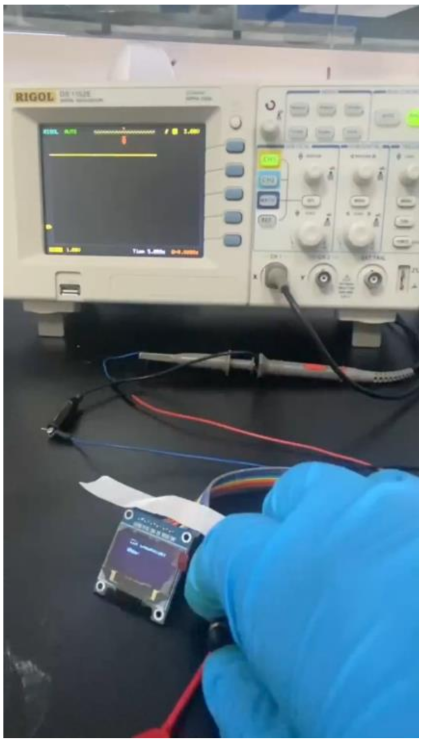

Video S3 S-P_3M_1hydrogel human-computer interaction test.

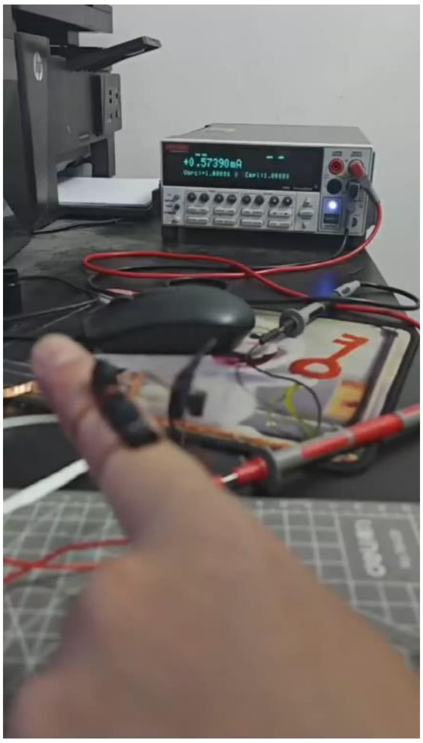

Video S4 S-P_3M_1hydrogel Morse code test.

Table S1. Comparison of mechanical properties of PVA/MXene type hydrogels.

Materials	Strain	Stress	Toughness	References
S-MP_3	450%	350 kPa	0.61 MJ/m^3	1
OMDDH	310%	1 MPa	----	2
PcNA-M	930%	70 kPa	----	3
MXene/PVA	170%	30 kPa	----	4
PCTM	740%	1.8 MPa	6.24 MJ/m^3	5
MSPPH	920%	180 kPa	810 kJ/m^3	6
S-P_3M_1	580%	2.87 MPa	9.92 MJ/m^3	This work

Table S2. Comparison of swelling resistance of S-PM hydrogels with other hydrogels.

Materials	Anti-swelling mode	Swelling ratio	References
DIPN	Double interpenetrating network	170%	7
PVA/NaCl/Amy	Dense hydrogen-bond network	30%	8
TA@Fe^{3+}-Cel-PAxMy	High density crosslinking	50±20%	9
HP(AAm/AA)–CS–Fe^{3+}	High density crosslinking	25%	10
P(AA-co-VBIBr)	Ionic complexations	100%	11
PDHO-US	Hydrophobic micelles	26%	12
S-P_3M_1	High density crosslinking	75%	This work

Table S3. Comparison of electrical properties of S-PM after swelling with other hydrogels.

Materials	Electrical properties or resistance change rate before swelling	Electrical properties or resistance change rate (%) after swelling	References
P(AA-MEA)-CS-Fe	0.33 S/m (original)	0.05 S/m (20h)	13
AA-IL	350% (Air)	275% (under water)	14
P(AA-SMA)-CMC-Na	0.06 S/m (original)	0.035 S/m (50h)	15
P(AA-LMA)$_{CTAB}$-Zr^{4+}-Eq	400% (original)	350% (7 days)	16
PABC	GF=2.14 (original)	GF=1.79 (5 days)	17
S-P_3M_1	333Ω<br>GF=0.28 (original)	353Ω<br>GF=0.73 (7 days)	This work

References

[1] Y. Yi, X. Chen, S. Feng, B. Chen, C. Lu, Z. Zhou, *Compos Part A-Appl S* **2023**, 175, 107793.

[2] Y. Liu, X. Lv, Y. Song, Q. Ao, B. Yuan, T. Huang, X. Tong, J. Tang, *ACS Sustainable Chem. Eng* **2023**, 11, 4177.

[3] F. Chen, H. Deng, G. Li, X. Li, J. Pan, T. Liu, T. Gong, *Chem Eng J* **2024**, 489, 151221.

[4] J. Wei, Y. Yang, F. Pan, K. Yang, Y. Wang, Z. Zeng, Q. Wang, Z. Fu, *Compos Part A-Appl S* **2023**, 172, 107626.

[5] D. Kong, Z. M. El-Bahy, H. Algadi, T. Li, S. M. El-Bahy, M. A. Nassan, J. Li, A. A. Faheim, A. Li, C. Xu, M. Huang, D. Cui, H. Wei, *Adv Compos Hybrid Ma* **2022**, 5, 1976.

[6] P. Li, H. Wang, Z. Ju, Z. Jin, J. Ma, L. Yang, X. Zhao, H. Xu, Y. Liu, *ACS Nano* **2024**, 18, 2906.

[7] W. Fan, L. R. Jensen, Y. Dong, A. J. Deloria, B. Xing, D. Yu, M. M. Smedskjaer, *ACS Appl Bio Mater* **2022**, 6, 228.

[8] Y. Gao, Y. Wang, Y. Dai, Q. Wang, P. Xiang, Y. Li, G. Gao, *Eur Polym J* **2022**, 164, 110981.

[9] J. Wang, J. Luo, Z. Jia, Y. Chen, C. Li, K. Zhong, J. Xiang, P. Jia, *ACS Appl Polym Mater* **2024**, 6, 6603.

[10] J. Xu, R. Jin, X. Ren, G. Gao, *J Mater Chem A* **2019**, 7, 25441.

[11] X. Ming, Y. Sheng, L. Yao, X. Li, Y. Huang, H. Zhu, Q. Zhang, S. Zhu, *Chem Eng J* **2023**, 463, 142439.

[12] S. Wang, L. Wang, X. Qu, B. Lei, Y. Zhao, Q. Wang, W. Wang, J. Shao, X. Dong, *ACS Appl Mater Interfaces* **2022**, 14, 50256.

[13] Z. Zhao, X. Qin, L. Cao, J. Li, Y. Wei, *Int J Biol Macromol* **2022**, 212, 123.

[14] L. Chen, X. Fei, Y. Zhou, J. Tian, L. Xu, Y. Li, *J Colloid Interf Sci* **2022**, 628, 287.

[15] Y. Cai, K. Wan, Q. Chen, M. Hong, Z.-X. Zhou, H. Fu, *J Mater Chem C* **2023**, 11, 12981.

[16] S. Pan, C. Xiang, Z. Liu, G. Tong, C. Zhang, X. Zhu, *J Mater Chem C* **2024**, 12, 8813.

[17] M. Qi, D. Zhang, Y. Guo, H. Zhang, J. Shao, Y. Ma, C. Yang, R. Mao, *J MaterChem A* **2024**, 12, 16839.
